# Genome Sequence of the Emerging Plant Pathogen *Dickeya solani* Strain RNS 08.23.3.1A

**DOI:** 10.1128/genomeA.01270-13

**Published:** 2014-01-30

**Authors:** Slimane Khayi, Samuel Mondy, Amélie Beury-Cirou, Mohieddine Moumni, Valérie Hélias, Denis Faure

**Affiliations:** a Institut des Sciences du Végétal, CNRS, Gif-sur-Yvette, France; b Université Moulay Ismail, Faculté des Sciences, Département de Biologie, Meknès, Morocco; c Comité Nord Plant de Pomme de Terre (CNPPT), Semences, Innovation, Protection Recherche et Environnement (SIPRE), Achicourt, France; d Fédération Nationale des Producteurs de Plants de Pomme de Terre (FN3PT-RD3PT), UMT Innoplant (FN3PT-INRA), Le Rheu, France

## Abstract

Here we present the genome sequence of *Dickeya solani* strain RNS 08.23.3.1A (PRI3337), isolated from *Solanum tuberosum*. *Dickeya solani*, recently described on potato cultures in Europe, is a proposed new taxon closely related to the *Dickeya dianthicola* and *Dickeya dadantii* species.

## GENOME ANNOUNCEMENT

Bacteria belonging to the *Dickeya* genus cause soft rot disease on a wide range of plants (1, 2), particularly on ornamentals (*Dianthus, Dahlia, Kalanchoe, Pelargonium, Chrysanthemum, Parthenium, Dieffenbachiae*, and *Saintpaulia*) and crops (chicory, banana, sunflower, rice, artichoke, pineapple, tomato, banana, maize, and potato). Infection is characterized by the maceration of plant tissues due to degradation of pectin, the major component of primary cell walls in plants (3). The resulting symptoms on potato stems are named blackleg and soft rot on tubers. On this plant host, disease symptoms caused by *Dickeya* spp. are similar to those caused by *Pectobacterium atrosepticum*. Three main *Dickeya* species, *D. dadantii*, *D. zeae* and *D. dianthicola*, were previously described on potato. A new taxon, tentatively named *D. solani*, has been recently identified largely in Europe and beyond (4). The *D. solani* strain RNS 08.23.3.1A (PRI3337) was isolated from potatoes in France in 2008 (5).

Here we report the *de novo* genome assembly of *D. solani* strain RNS 08.23.3.1A using Illumina HiSeq 2000 v3 technology. Two libraries were constructed using the TruSeq SBS v3 sequencing kit, a shotgun (SG) paired-end library with a fragment size of 150 to 500 bp and a long-jumping-distance (LJD) mate-pair library with an insert size of 6,000 bp. The two libraries were sequenced using the 2×100-bp paired-end read module by Eurofins Genomics (France). Assembly of the sequences was carried out by use of the software CLC Genomics Workbench (v5.1) from CLC bio. Sequence reads with low-quality (limit 0.05), ambiguous nucleotides (*n* ≤ 2) and sequence lengths of <30 nucleotides were dropped for assembly. In total, 5,682,625 mate-pair and 52,496,166 paired-end reads were obtained, corresponding to 419,377,725 and 4,903,141,904 bases, respectively. The average length was 73.8 bp for the mate-pair reads and 93.4 bp for the paired-end reads. The *de novo* assembly (length fraction, 0.5; similarity, 0.8) generated 42 contigs (>2,000 bp) with an average coverage of 1,075-fold. The average length of the contigs was 121,605 bp, and the largest contig was 483,425 bp, with an *N*_50_ contig size of 299,659 bp. Scaffolding of the contigs was processed using SSPACE basic v2.0 (6). Two scaffolds with gaps from 367 bp to 4,771 bp in size were obtained. For finishing, the gaps were closed by the mapping of mate pairs using as reference the 5 kbp from each of the contig ends (read length, 0.9; identity, 0.95). Then, using homemade script and fastqselect.tcl from the MIRA3 package, the mapped reads for both orientations (R1 and R2) were retrieved and *de novo* assembled (read length, 0.5; identity, 0.8). The remaining gaps were resolved by Sanger sequencing of PCR amplicons.

The assembled genome of *D. solani* strain RNS 08.23.3.1A is composed of one circular chromosome, containing 4,923,734 bp. The G+C content was 56.11%. Gene prediction using the RAST (Rapid Annotation using Subsystem Technology) v4.0 automated pipeline (7) revealed the presence of 4,337 open reading frames.

### Nucleotide sequence accession numbers.

This whole-genome shotgun project has been deposited at DDBJ/EMBL/GenBank under the accession number AMYI00000000. The version described in this paper is version AMYI01000000.
